# Retraction: JNK Contributes to the Tumorigenic Potential of Human Cholangiocarcinoma Cells through the mTOR Pathway Regulated GRP78 Induction

**DOI:** 10.1371/journal.pone.0310934

**Published:** 2024-09-18

**Authors:** The PLOS ONE Editors

After this article [1] was published, concerns were raised about results presented in Figs 1, 5, 6, and 7.

The concerns include:

- In the HCCC-9810 p-p70S6K panel in Fig 6A, lanes 3 and 4 appear similar.
- In the mTOR panel in Fig 6B, the lower part of the second lane appears similar to the lower part of the third lane.
- When levels are adjusted, there appear to be background discontinuities in some of the western blot panels in Figs 1, 5, 6, and 7.

The corresponding author stated that the underlying data are no longer available. In the absence of the underlying data, the concerns cannot be resolved. The issues call into question the integrity and reliability of the results reported in the above figures. Therefore, the *PLOS ONE* Editors retract this article.

RD did not comment on the retraction decision. CF, KH, CZ, SS, BL, YLi, CYD, SC, RC, YLiu, HL, MW, and XX either did not respond directly or could not be reached.
